# A facile synthesis of mono dispersed spherical silver doped bioactive glass nanoparticle

**DOI:** 10.1007/s10856-021-06496-9

**Published:** 2021-03-12

**Authors:** Zahra Kazemian, Mohammad Varzandeh, Sheyda Labbaf

**Affiliations:** Department of Materials Engineering, Isfahan University of Technology, Isfahan, 84156-83111 Iran

## Abstract

Bioactive glasses have attracted enormous attention in the field of biomaterials for dental and medical applications. Incorporation of antibacterial ions within BGs has been proved to be a promising approach to fortify their bactericidal character. In this study, homogenous BGs containing silver (Ag) ions were synthesized by sol–gel method. Subsequently, the presence of the embedded ions were characterized by X-ray fluorescence (XRF) elemental analysis and energy dispersive X-ray (EDX) spectroscopy. Moreover, released ions were measured in simulated body fluid (SBF) and their antibacterial effectiveness was further verified using minimum bactericidal concentration (MBC) and minimum inhibitory concentration (MIC) tests. A crystalline hydroxyapatite layer was formed on the Ag-BG surfaces at day 5 approved by X-ray diffraction indicating the preserved bioactivity. The resultant uniform, mono-dispersed and dense nanoparticles show 19 great potential for a range of orthopedic and dental applications.

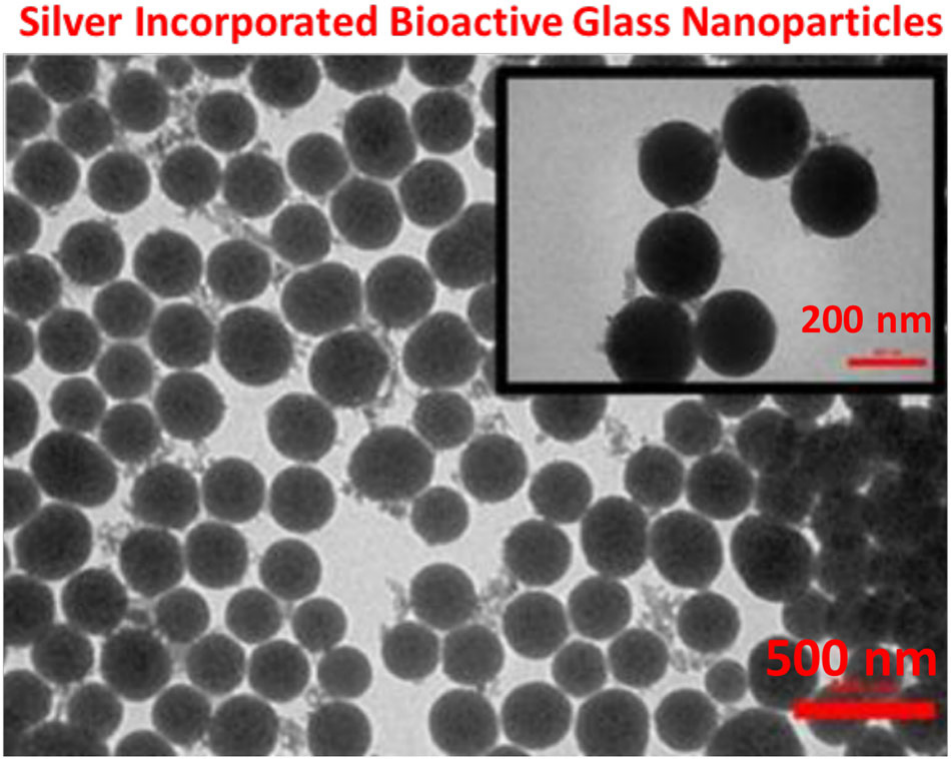

## Introduction

The ion-dependent tunable biodegradability and osteogenic behavior of bioactive glasses (BGs) have made them ideal candidates for orthopedic applications [1–3]. Previous studies have shown the in vitro antibacterial activity of SiO_2_ 53% Na_2_O 23% CaO 20%–P_2_O_5_ 4% (S53P4) BG by increasing the pH of bacteria microenvironment and osmotic pressure [4] caused through the release of ionic dissolution products leading to the damaging of bacterial cell wall by BG sharp debris [5]. However, in-vivo blood flow and buffering system diminished this property [6]. Therefore, there has been a great shift towards multifunctional BG structures with an improved intrinsic antibacterial property [7]. The inspiring biological role of BG dissolution products such as P [8, 9], Ca[10], and Si [11, 12] on osteoblast proliferation, differentiation, and ECM mineralization is well known. However, through the utilization of BGs for in vivo applications, the high risk of infection still remains a challenge. Previous studies have shown that ions like Cu [13–15], Zn [16], Ag [17], Ce [18], Ga [19], and Mn [20] have been incorporated into BG for combating the bacteria.

Amongst all, silver ion acts as an effective antibacterial agent via interaction with the thiol groups of proteins and cause DNA dysfunction in gram positive and gram negative bacterium [21]. Apart from that, using antibacterial silver nanoparticles itself cause toxicity by generating reactive oxygen species [22] and cause mitochondria dysfunction [23] for adjacent cells which has, hence, restricted its clinical application. Also, silver particle-specific antibacterial is negligible [24]. So, the researchers have chosen the Ag^+^ ion dominantly against bacterium.

Many attempts have been made to incorporate Ag ions into the BG structure using sol–gel method [25] and molten salt ion exchange method [26]. Zhu et al. [17] investigated the role of amine modification on Ag^+^ loading in mesoporous 58 S (SiO_2_ 58%–Na_2_O 33%–P_2_O_5_ 9%). They observed enhanced Ag^+^ loading of 5.29% which resulted in improved antibacterial behavior after 768 h post testing. In a different study, Palza et al. [25] synthesized BG microparticles containing Ag^+^ and Cu^2+^ ions using sol–gel method.. Minimum concentration of Ag-BG (1 mg/ml for *E. coli* and 3 mg/ml for *S. mutans*) was lower than Cu-BG (125 mg/ml for *E. coli* and 8 mg/ml for *S. mutans*) in minimum bactericidal concentration (MBC) testing.

In the current study, spherical and mono-dispersed sub-micron Ag containing bioactive glass particles are prepared through a simplified sol–gel method to evaluate its suitability for bone related applications. The uniform and mono-dispersed particles could have numerous biomedical application. BG nanoparticles could be directly injected into the defect site and could be internalized by cells for the local sustained delivery of inorganic therapeutic ions. The particles also have great potential to be incorporated within a polymeric matrix to create a nanocomposite scaffold for bone tissue engineering or be coated on a bio metallic substrate to increase corrosion resistance and enhance surface bioactivity.

## Materials and methods

### Synthesis of nanoparticles

Monodispersed BGs sub-micron particles were prepared by modifying our previous synthesis process [27]. Briefly, ethanol:deionized water with volumetric ratio of 9:1 was prepared. 100 μLof tetraethylorthosilicate was added to 5 mL of the above-mentioned solvent and left for 20 min sonication before 5 ml sodium hydroxide was added. 0.045 g and 0.11 g of calcium nitrate and silver nitrate were added, respectively with 45 min time intervals. Resultant particles were centrifuged and then dried in oven over night. Finally, the sintering procedure was applied at 600 °C which heating and cooling rate was 5 °C/min and 10 °C/min.

### BG characterization

The morphology of the synthesized NPs was analyzed by Scanning Electron Microscopy (SEM- PHILIPS XL30) and transmission electron microscopy (TEM, JEOL. Composition analysis was conducted using of the Energy Dispersive Spectroscopy (EDS) and X-ray fluorescence (XRF) techniques. Phases and crystallinity of the samples were characterized by X-ray diffraction (XRD, X’Pert Pro X-ray diffractometer, Phillips, Netherlands). Image J software was utilized to measure particle size.

### Bioactivity testing

The apatite formation ability of Ag-BG was assessed in accordance to our previous study [27]. First, the samples were soaked in the simulated body fluid (SBF) at 2 mg/ml concentration at 37 °C for 1 and 3 days. The pH of the solution was assessed throughout the testing. At the end of each time point, particle was separated, washed and dried in oven at 40 °C overnight. XRD was conducted to characterize the formed apatite layer. To evaluate degradation through ionic release, inductively coupled plasma (ICP) was conducted.

### Antibacterial activity

Antibacterial activity of Ag-BG NPs was assessed using minimum inhibitory concentration MIC and MBC tests. The assays were conducted against standard *Staphylococcus aureus ATCC 6538* and *Escherichia coli ATCC 25922* as gram-positive and gram-negative bacteria, respectively. A concentration range of 35–30,000 μg ml^−1^ was selected. All tests were conducted in triplicates and according to clinical and laboratory standard institute (CLSI) [28].

### Cell culture

The human osteoblast cells (MG63) were obtained from the Pasteur Institute (Iran) and were cultured in Dulbecco’s modified eagle’s medium-high glucose12800 (DMEM12800), fetal bovine serum, penicillin, streptomycin, glutamax, and trypsin/EDTA solutions were obtained from Gibco (Germany). Cells were seeded at a density of 10,000 cells/well in a 48 well/plate. Samples were heat-sterilized at 120 °C for 2 h followed by UV sterilization and prepared at 100 and 250 µg/ml concentrations. Prior to cell exposure the resultant suspensions were sonicated for 15 min followed by vortex to ensure particle dispersion. At the end of each time point (1, 3, and 5 days) MTS reagent was added to each well and incubated for 3.5 h at 37 °C and measured at 450 nm using a microplate reader (Bio-Rad, USA).

## Results and discussion

**Fig. 1:**
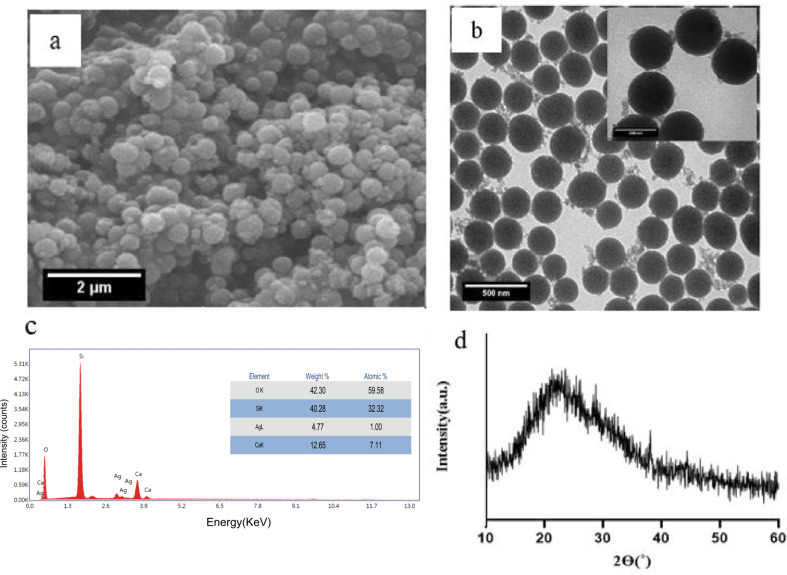
**a** SEM and **b** TEM images (scale bar 500 nm and inset 200 nm) of Ag-BG with inset showing a higher magnification image, **c** the corresponding EDS analysis, and **d** XRD pattern of particles calcinated at 600 °C

Ag doped BG (Ag-BG) nanoparticles is presented in Fig. 1a,b. According to images, uniform and monodispersed particles have been synthesized using a facile processing method. Also, the nanoparticles exhibited more smooth and regular shape in comparison with the previous works [29]. An average particle size of 200 ± 25 nm was obtained (*n* = 50), which is significantly smaller than a previous study where 400 nm Ag-BG particles were produced [30]. Figure 1c confirms the presence of Si, Ca, and Ag in the BG particles. The XRD spectra in Fig. 1d show a broad peak within 15–30 corresponding to the glassy state of the particles. This is in good agreement to our previous studies [27, 31].

The accuracy of the Ag ion doping into the BG was further evaluated using XRF. Regarding to the XRF elemental analysis results in Table 1, the SiO_2_, CaO, and Ag concentration of examined was found to be 74, 24, and 2 wt%, respectively. The molar fraction resulted from XRF confirms the EDX results with some variations. In the other words by overlooking the oxygen fraction in EDX results the molar fraction of Si^4+^, Ca^2+^, and Ag^+^ equals to 80, 17.5, and 2.5%, which is close to the results emanated from XRF. This statement is now added to the revised manuscript.

**Table 1:** XRF elemental analysis of as-prepared Ag-BG particles

Compound	SiO_2_	CaO	Ag_2_O
Concentration (%wt)	74	24	2

Our observations suggest that BGs dissolve in SBF, changing the pH of the surrounding medium by releasing ionic products liberated from BG particulates. Dissolution from the BGs leads to a super-saturation of Ca ions in the SBF solution and subsequent re-precipitation of Ca and P rich crystals on their surface. Dissolution of the silica network (breaking Si–O–Si bonds) then occurs. Ca and P can combine in solution and deposit onto silanol bonds on the glass surface, nucleating a hydroxycarbonate apatite layer.

**Fig. 2:**
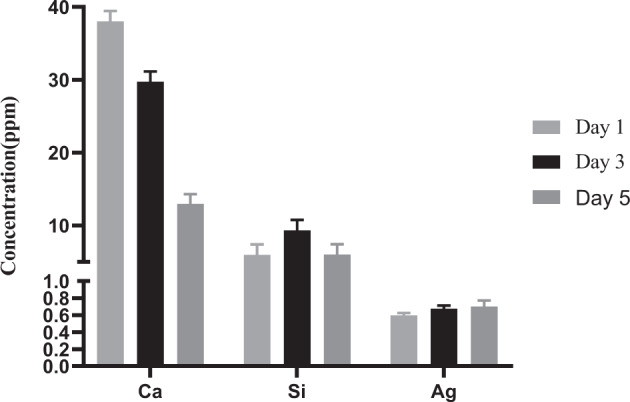
ICP analysis of the Calcium (Ca^2+^), Silicon (Si^4+^), and Silver(Ag^+^) ions release in the medium containing Ag-BG at day 1, 3, and 5

Sol–gel derived BGs are known to have a greater rate of bioativity, through dissolution and the release of ionic product, owed to their high surface and high level of surface OH groups. Here, the dissolution of Ag-BG structures through ionic release study following immersion in SBF was evaluated by ICP following 1, 3, and 5 days (Fig. 2). Our observation suggests a supersaturation of Ca ions in the SBF solution, which subsequently results in re-precipitation of Ca and P (from SBF) crystals on Ag-BG surface. The dissolution of Si–O–Si network then occurs, creating silanol bonds onto which Ca and P in the form of apatite can deposit. The Ag ion amount within solution has increased from day 1–3 similar to a previous report [17]. The maximum Ag ion release was in day 5, However, a decreasing trend in Ca was observed.

**Fig. 3:**
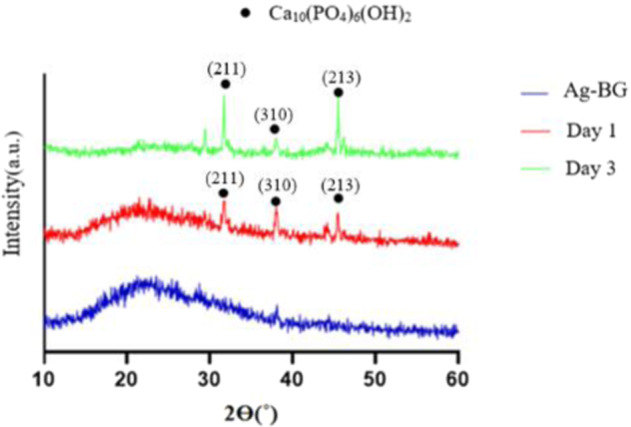
**a** XRD patterns of the Ag-BG particles following immersion in SBF after at 1 and 3 days

In order to prove the Ag-BG bioactivity, hydroxy apatite (HA) formation on their surface after SBF immersion was evaluated by the means of XRD and EDS. As illustarted in Fig. 3, XRD patterns are gradually transforming from amorphous structure to the crystalline structure [32]. The narrow peaks at 31.9, 39, and 48 illustrate the highly crystalline HA phase formation at 1 and 3 days. The peaks of HA deposition detected after 1 day, have sharpened by day 3. This is an important finding as it highlights the enhaced bioactivity rate of Ag-BG nanoparticles compared to a previous study on Ag-BG [30].

The antibacterial activity of the Ag-BG particles against gram positive and gram negative bacteria was evaluated by MBC and MIC tests. The bactericidal and inhibitory concentration of the Ag-BG against *s. Aureus* was half of that of *E. Coli*. As shown in Table 2, the MBC and MIC concentration of the Ag-BG particles was 134 μg/ml and 268 μg/ml, respectively. This concentration is moderately lower than the previous studies which provides higher efficiency [14]. The amorphous structure of the present study instead of crystalline structure, enhanced the available Ag ions for effective bactericidal activity [25, 29, 33].

**Table 2:** MBC and MIC antibacterial test of Ag-BG against *Staphylococcus aureus* and *E.coli*

Bacteria	MBC (µg/ml)	MIC (µg/ml)
*S. aureus* ATCC 6538	134	134
*E.coli* ATCC 25922	268	268

**Fig. 4:**
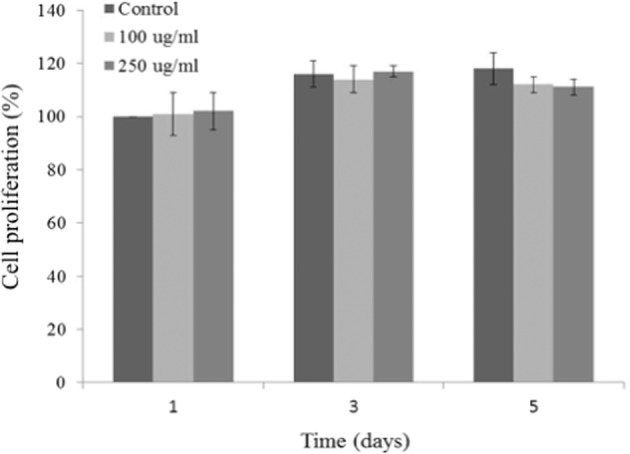
MTS cell viability assay following 1, 3, and 5 days for Ag-BG tested at 100 and 250 µg/ml. The data represent the mean values ± SD of two individual experiments each performed in triplicate and are normalized to the control of day 1

Cell metabolic activity and consequently cell viability was assessed using MTS (Fig. 4). As can be seen, cell viability has not been affected by the addition of Ag-BG nanoparticles, except a reduction at a higher concentration of 250 µg/ml, after 5 days of culture, is detected. This finding has previously been reported [14] for SiO_2_–CaO BG nanoparticles and was attributed to the mechanism of calcium homeostasis by the mitochondria, hence influencing metabolic activity and cell viability results.

## Conclusion

Monodisperse Ag-BG particles was successfully synthesized through a simple sol-gel method with simultaneous bioactivity and antibacterial behavior. The bioactivity of the Ag-BG was confirmed by XRD characterization of hydroxy-carbonate apatite layer formation on their surfaces following the immersion in SBF. Amount of released Ag ions in SBF have measured by ICP which was sufficient for antibacterial activity as further evidenced by MBC and MIC tests. Particle did not inhibit the cellular growth pattern and were found to be non-cytotoxic.

## Supplementary information

Supplementary Figure
