# Supplementary material for: A facile synthesis of mono dispersed spherical silver doped bioactive glass nanoparticle
Source: J Mater Sci Mater Med. 2021 Mar 12;32(3):29. doi: 10.1007/s10856-021-06496-9 (PMC7952368; doi:10.1007/s10856-021-06496-9)
Supplement: Supplementary file 1 — Supplementary Figure [file 10856_2021_6496_MOESM1_ESM.docx]

**Supplementary Figure:**


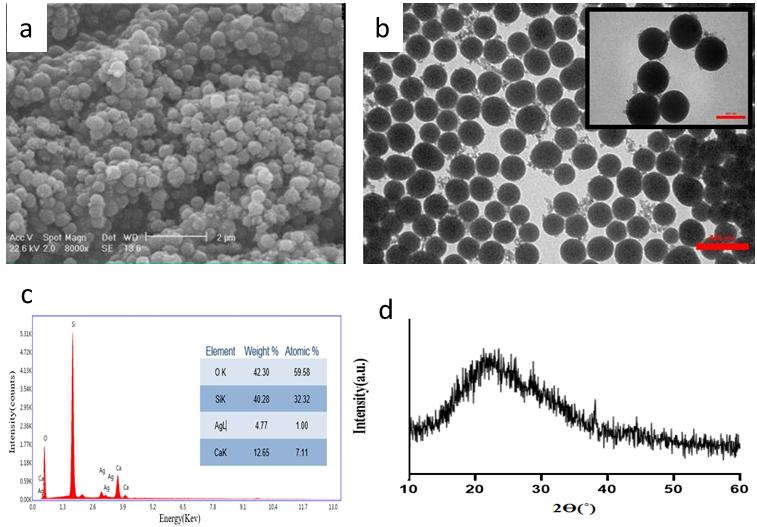

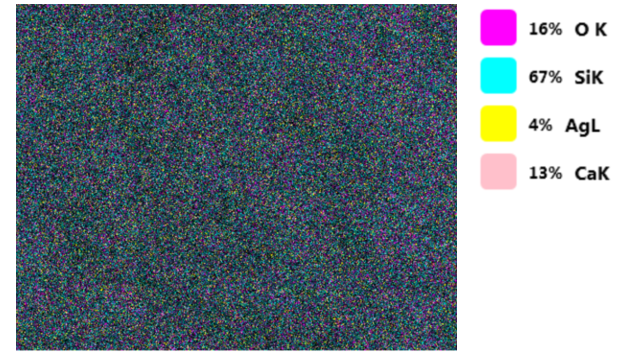

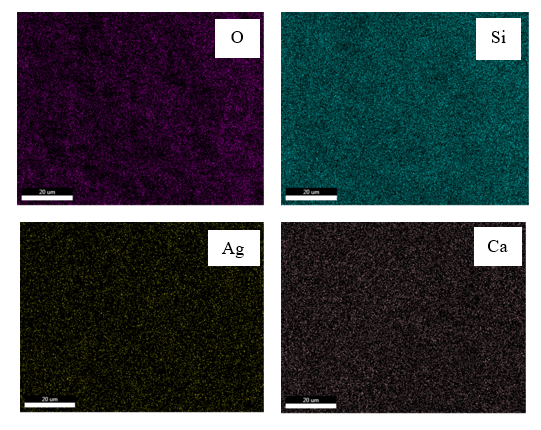


**Supplementary 1:** SEM-EDS mapping of the O, Si, Ag and Ca elements in as-synthesized Ag-BG.
